# Supplementary material for: Evaluation of oral dextrose gel for prevention of neonatal hypoglycemia (hPOD): A multicenter, double-blind randomized controlled trial
Source: PLoS Med. 2021 Jan 28;18(1):e1003411. doi: 10.1371/journal.pmed.1003411 (PMC7842885; doi:10.1371/journal.pmed.1003411)
Supplement: Attachment — Submitted filename: Response to reviewers23July2020.docx [file pmed.1003411.s002.docx]

**Response to reviewers’ comments.**

Neonatal hypoglycemia prevention with oral dextrose gel (hPOD): A multicenter, double blind, randomized controlled trial." (PMEDICINE-D-20-01227R1)

Requests from the editors:

*Per the journal's usual style, please include (ideally in the last sentence of the Abstract Methods and Findings section), a brief note about any key limitation(s) of the study's methodology.

*This has been added as requested (Abstract, lines 42-43)*

*At this stage, we ask that you include a short, non-technical Author Summary of your research to make findings accessible to a wide audience that includes both scientists and non-scientists. The Author Summary should immediately follow the Abstract in your revised manuscript. This text is subject to editorial change and should be distinct from the scientific abstract. Please see our author guidelines for more information: <https://journals.plos.org/plosmedicine/s/revising-your-manuscript#loc-author-summary>

*This has been added as requested (page 3)*

*Ideally, please reformat the in-text citation callouts so these are sequential numerals in square brackets (eg, [1], [2] etc) rather than superscript numerals - if using referencing software this should be quick and easy.

*References have been reformatted as requested*

-----------------------------------------------------------

Comments from the reviewers:

Reviewer #1:

This review relates to manuscript PMEDICINE-D-20-01227R1 about the results of hPOD trial.

Overall, I found the manuscript well written and easy to follow. I only have minor comments listed below:

*	Babies for whom the primary outcome was not available were assumed to have been admitted to NICU (conservative analysis), but there was no other imputation for missing data. In the results, please clarify how many babies in each arm were subject to that imputation. Could a different, potentially more extreme, imputation scenario have led to different results?

*All three babies were in the placebo group. This is noted in the results (lines 205-6). We pre-specified this conservative approach without knowledge of group allocation. We also note that omitting these babies from the analysis did not change the findings (see modified per protocol analysis, Table 3).*

*A more extreme imputation scenario would be to allocate all of these babies to no NICU admission. This also does not change the findings (aRR 1.13, 95% CI 0.88,1.46 compared to 1.10 (0.85,1.42) in the current analysis). Because it was not pre-specified, we have not included this in the manuscript.*

*	When describing the variables used to adjust the primary analysis model (lines 130-132), please clarify which variables entered as fixed vs random effects. Please also clarify the reason for modelling the maternal unique identifier as a clustering term (presumably due to multiple babies from a single mother) together with the method used to do so (e.g. generalised estimating equations or random effect).

*These points have been clarified in the methods section (lines 162-3). Yes the use of maternal identifier as a clustering term was to account for the non-independence of multiple babies with the same mother. This has also been clarified in the methods section (line 163).*

*	The exploratory analysis section (lines 135-139) mentions adjustments by centre and maternal unique identifier. These terms also appear to be included in the primary analysis described in the preceding paragraph. Please clarify whether adjusting for centre and adding the maternal unique identifier was pre-specified vs post-hoc and whether it was applied to all analyses vs only exploratory ones.

*The adjustments were pre-specified in the statistical analysis plan, and were applied to all analyses. This is now clarified in the results (line 160).*

*	The design section mentions 16 sites whereas the results section starts by mentioning 18 sites. Please clarify / correct.

*We apologise for this error. There were 18 sites, as correctly stated in the results. The number has been corrected in the study design section (line 97).*

*	I note that the SAP is dated August 2019 which is after recruitment ended (May 2019). Please confirm that it was written and finalised by authors who were unaware of the trial results.

*We confirm that the SAP was written and finalised by the Steering Group before the investigators were aware of any trial results.*

*	The exploratory subgroup analyses by centre (lines 205 - 209) mentions results with "data not shown". Please note that it is the journal's policy that every result discussed should be included either in the manuscript or the online supplement. Please note that the same applies to exploratory results reported on lines 229-230.

*The additional data are now provided in Figure 2 and in the text (lines 270-271)*

*	When presenting the results of the subgroup analyses, please consider reporting the p-value for heterogeneity (that is the significance of the interaction term between the subgroup variable and the intervention) instead of the p-value testing the treatment effect within each subgroup. Please also consider using a forest plot in place of a table to present (some of) the subgroup analyses.

*The subgroup analyses are now provided in Figure 2, and the interaction terms are included.*

*	Please clarify the hypothesis being tested by the analysis presented in Figure 2 (relationship between the effect of dextrose gel on NICU admission and a) rate of NICU admission and b) rate of hypoglycaemia).

*Our hypothesis was that the effect of dextrose gel on these variables may be influenced by the underlying incidence ie it may have a greater or more readily detected effect in centres where the rate of NICU admission or the rate of hypoglycemia was high, but minimal or no effect if these rates were already low. Note that this is now figure 3.*

*	In the methods section, please indicate which subgroup analyses were pre-specified (i.e. planned before seeing any result by treatment arm) vs post-hoc (conducted after seeing results).

*All analyses were prespecified, with the exception of the exploration of the relationship between mode of delivery and initial blood glucose concentration, which is stated in the results as a post hoc analysis (line 256). This has been clarified in the methods (line 170).*

*	The attached SAP mentions a longitudinal analysis of blood glucose measurements using a repeated-measure linear mixed model. I would like to see the results of this analysis included in the paper and presented using a longitudinal mean plot accompanied by the mean difference in blood glucose obtained from the mixed model.

*These analyses are now included in the methods (lines 171-5) and results presented in lines 177-9 and Figure 4.*

-Laurent Billot

-----------------------------------------------------------

Reviewer #2: This study builds on the previous work of Drs. Harding and colleagues. Neonatal hypoglycemia remains a significant and common health problem in the neonate and there is considerable controversy regarding treatment and neurological/developmental outcomes. In their previous landmark study this group showed that neonatal hypoglycemia in at risk populations was far more prevalent than previously appreciated and that even relatively mild hypoglycemia was associated with adverse outcomes in childhood. Dextrose gel is now widely used as a treatment for hypoglycemia but it remains unknown whether or not prophylactic treatment will prevent the development of hypoglycemia and whether that matters. Thus, this current study is a critical first step to address these questions. There are a few minor issues that should be addressed as outlined below.

1. It would be helpful to the general reader to define neonatal hypoglycaemia

*A definition has been added to the abstract (line 37) and is stated in the methods (line 137)*

2. I am not sure it is useful to include the mean birthweight of the entire group (line 149) as BW were different between the groups (LGA, SGA IDM, etc). It would be more helpful to this reviewer to instead have the average weights of each group.

*Mean weights of each group have been added (lines 186-7)*

3. Were there any differences between centers in numbers of babies in each group (SGA, LGA, etc)? I am not certain as to whether these are important data to include, but given the differences in admission to NICU between centers, it might be helpful to include.

*The distribution of risk factors did vary across centers, although some centers were very small. However, infants of women with diabetes comprised the largest group for every center. Since we have adjusted for recruiting center and risk factor in all analyses, we do not think this is likely to have influenced our results, and have chosen not to include these additional data in the manuscript.*

-----------------------------------------------------------

Reviewer #3: This is a very well designed double blind randomized controlled clinical trial. The study design, methods and results are superbly presented.

The aim was to assess whether prophylactic dextrose gel given to babies at risk of neonatal hypoglycemia (NH) would reduce admission to an NICU. The investigators have clearly accomplished their aims.

My comments are all rather minor.

Blood glucose concentrations were measured at 2 hours of age (i.e., 1 hour after receiving dextrose) and thereafter according to hospital standard practice for monitoring babies at risk of hypoglycemia.

I presume the policies were not identical at all 16 participating institutions. The more frequently one measures blood glucose ("how frequently it is sought"), the greater the chance hypoglycemia will be detected. I wonder to what extent any such differences may have influenced the frequency of detection of biochemical hypoglycemia. This is discussed on P16 as a limitation of the "pragmatic multicenter trial".

*We agree that this is of interest, which is why we undertook the exploratory analyses presented in figure 3, and discussed the issue as noted.*

Comments

P4 line 83 a minor point:  the diabetes community is on a campaign to stop referring to people with diabetes as "diabetic"

Infant of a mother with diabetes

*Noted and corrected throughout*

By far the most common reason for risk of hypoglycemia was maternal diabetes (type 1, type 2 and gestational diabetes). The authors comment on this in the discussion (on P. 17).

Neonatal hypoglycemia in these infants is thought to be caused by dysregulated insulin secretion by beta cells exposed to hyperglycemia in utero. The cellular processes that eventually lead to normal beta cell function after delivery presumably take time (several hours or days) to become normal. For this reason, I am not surprised that a single oral dose of glucose per se had only a modest effect to prevent subsequent hypoglycemia caused by dysregulated insulin secretion. Indeed, oral glucose might be expected to induce insulin secretion and, in the absence of adequate enteral nutrition (carbohydrate, protein and fat) might lead to so called reactive hypoglycemia?

Likewise, although the data are less robust, it is thought that dysregulated insulin secretion may play a role in the hypoglycemia of SGA and LGA babies.

*We recognize this theoretical concern, although we have shown both in this manuscript and previous publications on the use of dextrose gel that there is no evidence of reactive hypoglycemia, at least as indicated by the frequency and severity of hypoglycemia detected in infants randomized to receive dextrose gel vs placebo.*

-----------------------------------------------------------
