# Supplementary material for: Evaluation of oral dextrose gel for prevention of neonatal hypoglycemia (hPOD): A multicenter, double-blind randomized controlled trial
Source: PLoS Med. 2021 Jan 28;18(1):e1003411. doi: 10.1371/journal.pmed.1003411 (PMC7842885; doi:10.1371/journal.pmed.1003411)
Supplement: Attachment — Submitted filename: Response to Editorial Comments27Nov2020.docx [file pmed.1003411.s003.docx]

Response to Editorial Comments

1. Abstract:

a. Please correct the lower CI bound here to match Table 2 (0.86): “NICU admission occurred … (aRR 1.10, 95% CI 0.85,1.42, p = 0.44).”

*Corrected as requested*

b. Please clarify here that 95% would recommend the study to friends: “Maternal satisfaction was high (95%).”

*Reworded as requested*

2. Please ensure references in the bibliography follow the Vancouver style: first six authors should be shown before “et al.”, eg for reference 7.

*This reference has been corrected, and references 13 and 14 have also been updated*
